# A Systematic Treatise on the Injuries and Surgical Diseases of the Scalp, Skull, and Brain and Its Membranes

**Published:** 1861-01

**Authors:** 


A SYSTEMATIC TREATISE ON THE INJURIES AND SURGICAL DISEASES OF THE SCALP, SKULL, AND BRAIN AND ITS MEMBRANES.—The senior editor of the REVIEW begs leave to announce to his professional brethren that he is now engaged upon the composition of this work, and he earnestly and respectfully requests their co-operation in its construction, by furnishing him with such cases and practical reflections as may have arisen in the course of their experience. His object will be to present a complete digest of the literature upon the subject, and he trusts that he will thus be enabled to produce a work that shall be of permanent value to the healing art. Communications should be sent in at an early day. American journals will confer a favor by copying this notice.

